# Supplementary material for: Impact of global climate cooling on Ordovician marine biodiversity
Source: Nat Commun. 2023 Oct 10;14:6098. doi: 10.1038/s41467-023-41685-w (PMC10564867; doi:10.1038/s41467-023-41685-w)
Supplement: Supplementary file 1 — Supplementary Information [file 41467_2023_41685_MOESM1_ESM.pdf]

Supplementary Information

Impact of global climate cooling on Ordovician marine biodiversity

Daniel Eliahou Ontiveros[1*], Gregory Beaugrand[1], Bertrand Lefebvre[2], Chloe Markussen Marcilly[3], Thomas Servais[4], Alexandre Pohl[5*]

[1] Univ. Littoral Côte d'Opale, CNRS, Univ. Lille, UMR 8187 LOG, F-62930 Wimereux, France

[2] Univ Lyon, Univ Lyon 1, ENSL, CNRS, LGL-TPE, F-69622, Villeurbanne, France

[3] Centre for Earth Evolution and Dynamics, University of Oslo, 0315 Oslo, Norway

[4] Univ. Lille, CNRS, UMR 8198-Evo-Eco-Paleo, F-59000 Lille, France

[5] Biogéosciences, UMR 6282 CNRS, Université de Bourgogne, 6 Boulevard Gabriel, 21000 Dijon, France.

*Corresponding authors: danyeo@hotmail.fr, alexandre.pohl@u-bourgogne.fr

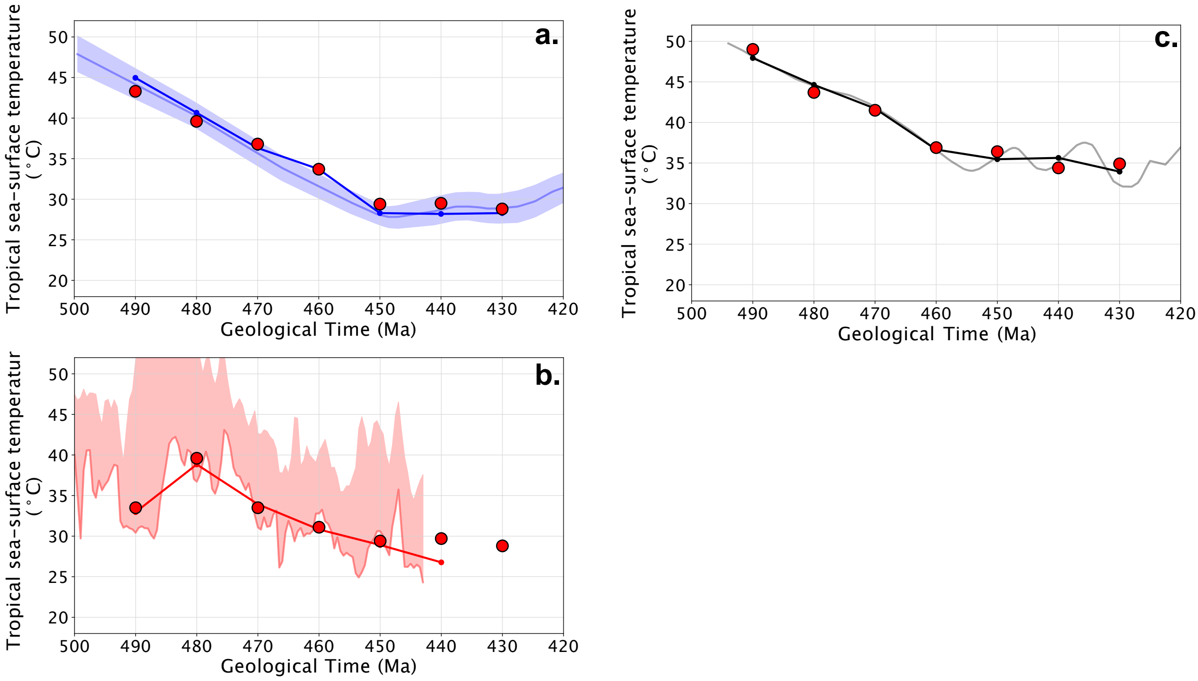

Supplementary Fig. 1. Ocean temperatures during the GOBE. Proxy-derived (curves, sometimes with envelopes) and simulated (large red dots) tropical (30 °N–30 °S) sea-surface temperatures in our (a) main cooling scenario[1], (b) alternative cooling scenario #1[2], and (c) alternative cooling scenario #2[3]. In each panel, the proxy-derived temperature trends published originally are shown with the thin curve (and envelope if applicable). The thick line with small dots represents the same data averaged in 10-Myr time bins (i.e., at our model resolution). For each time slice of each scenario, FOAM simulations (large red dots) were chosen to best fit sea-surface temperatures reconstructed based on proxy data. In scenario (a), data from ref.[1] were modified to represent only low-latitude temperatures after ref.[4]. In scenario (b) the lowermost temperatures of the envelope are supposedly the most robust (i.e., less diagenetically altered)[2]; they are used to derive the 10-Myr average. For 440 and 430 Ma in panel (b), in the absence of proxy data, climatic simulations we selected to display a plateau as in the (temporally more complete) datasets of panels (a) and (c).

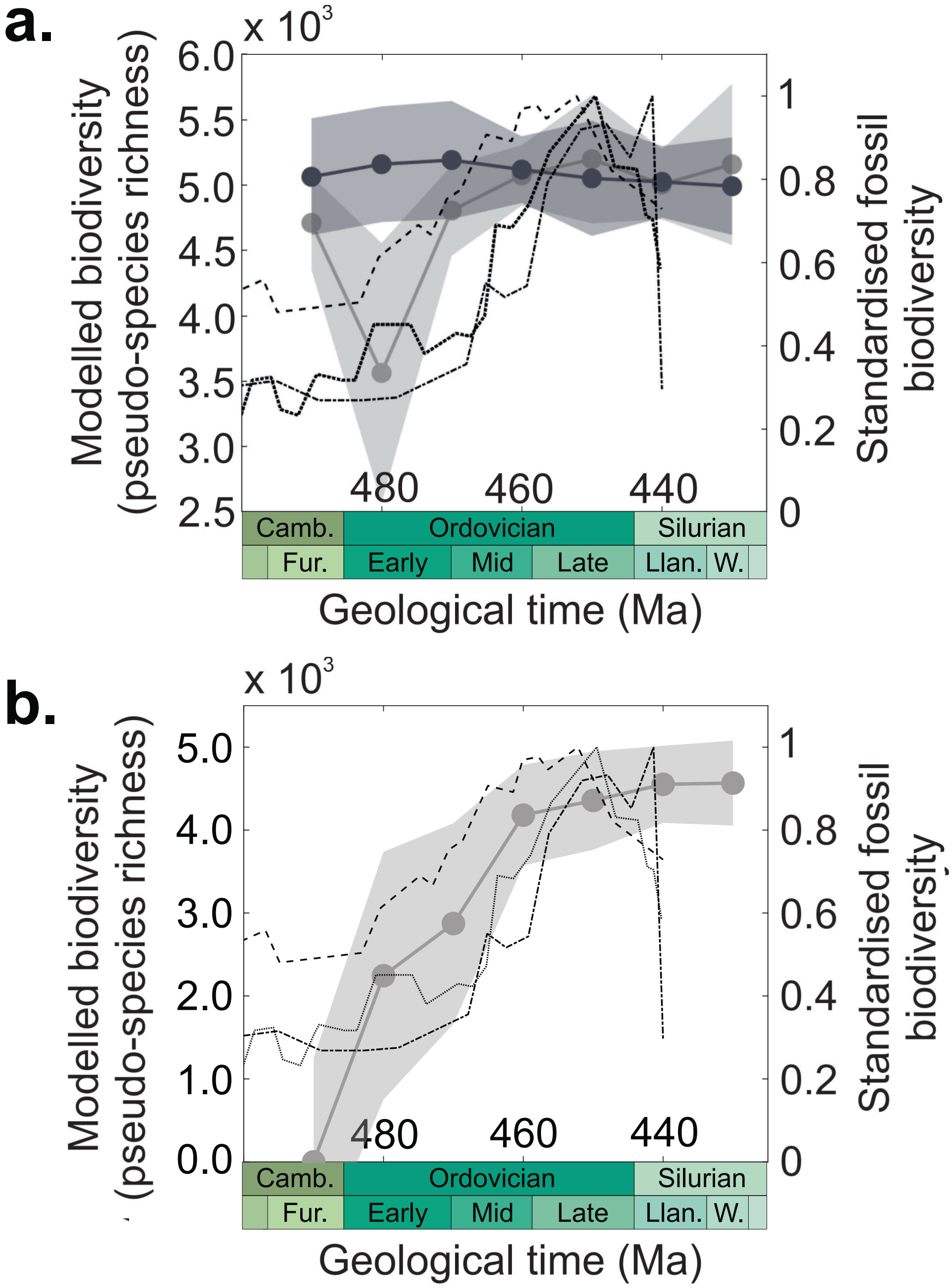

Supplementary Fig. 2. Impact of alternative cooling scenarios. As per Fig. 2 but using the temperature scenarios after (a) ref.[2] and (b) ref.[3]. Camb.: Cambrian; Fur.: Furongian; Llan.: Llandovery; W.: Wenlock.

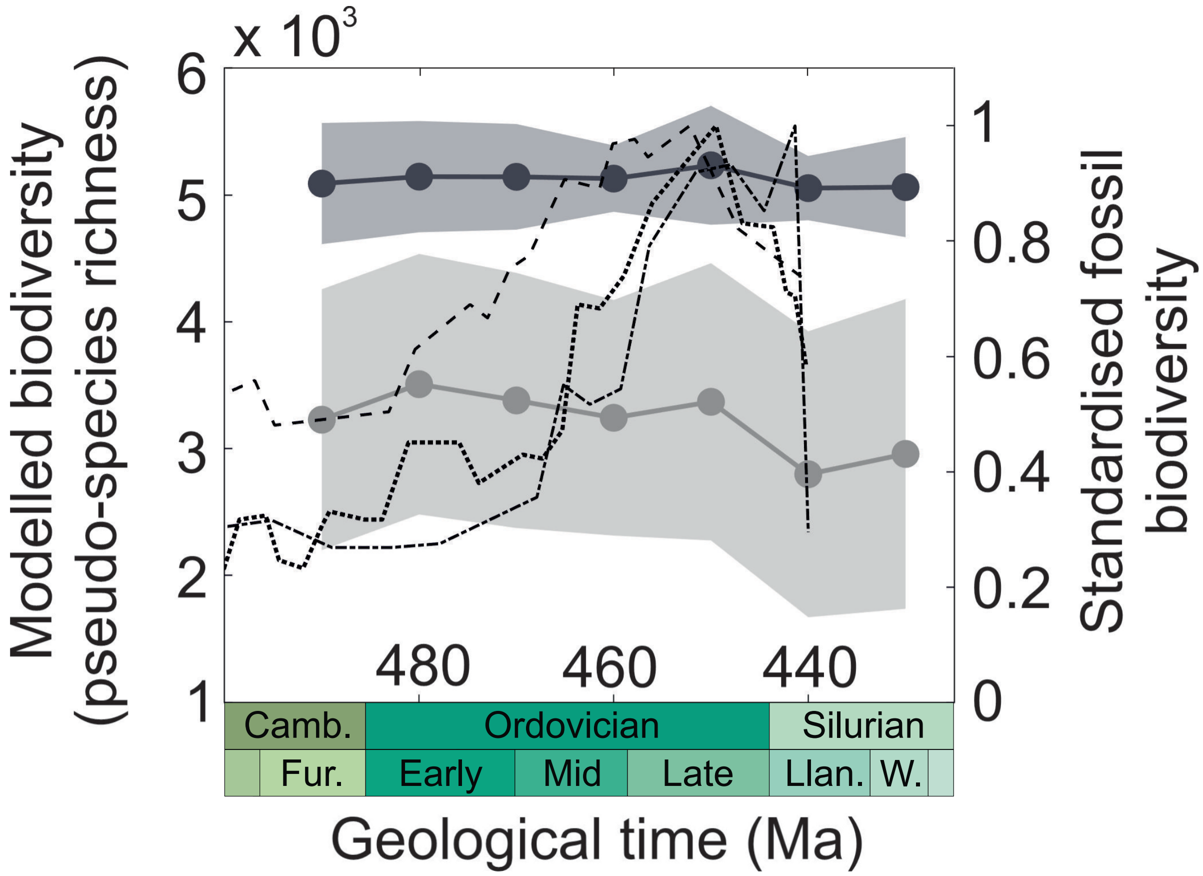

Supplementary Fig. 3. Contribution of the paleogeographical evolution to simulated biodiversity trends. As per Fig. 2 but using constant climatic states characterized by tropical sea-surface temperatures of ca. 40 °C (light gray) and 30 °C (dark gray). Camb.: Cambrian; Fur.: Furongian; Llan.: Llandovery; W.: Wenlock.

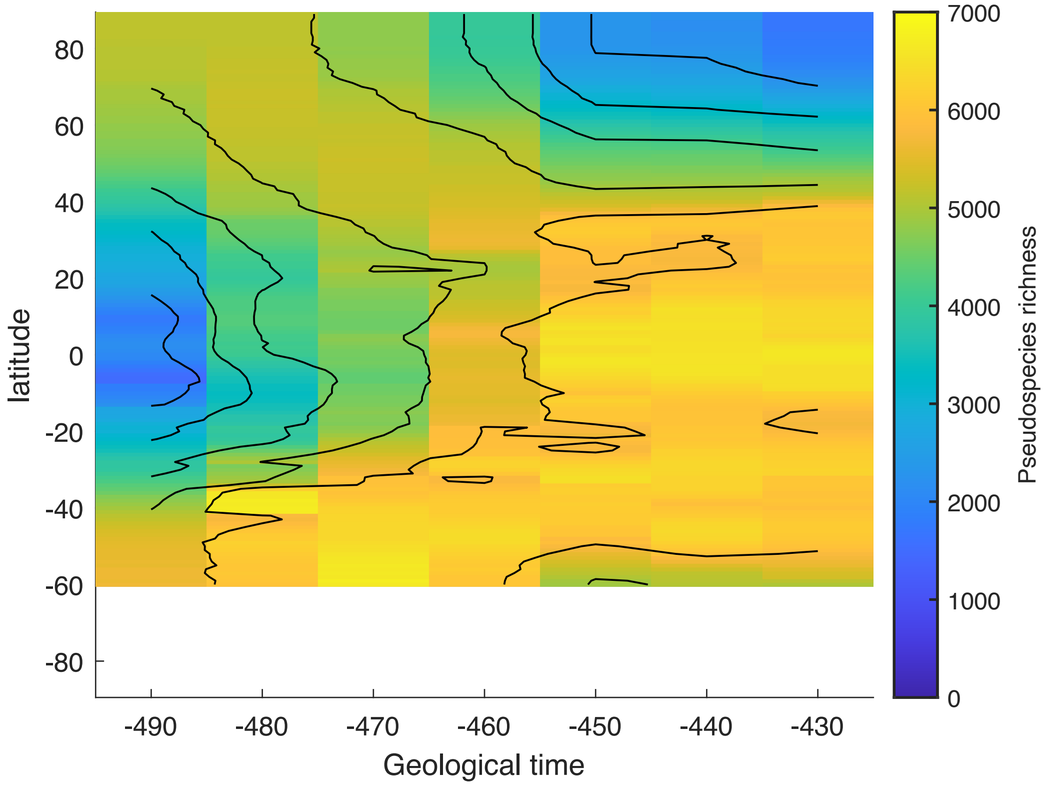

Supplementary Fig. 4. Sensitivity of the evolution of the simulated latitudinal biodiversity gradient to model spatial domain. As per Fig. 4, but with pseudo-species able to settle in the global ocean (instead of the shallow-water shelves only).

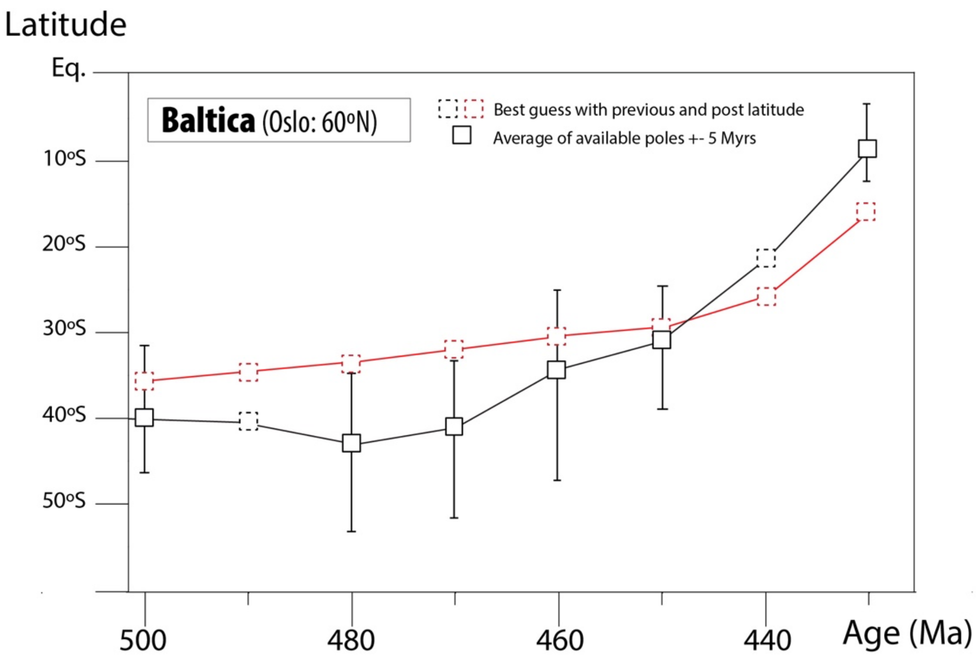

Supplementary Fig. 5. Uncertainty in the latitudinal position of Baltica (Oslo) during the Ordovician. 95 % confidence interval for the latitudinal position of Baltica during the Ordovician in the reconstructions of ref.[5] (black line with error bars). This model is based on paleomagnetic input poles which are fitted with spherical splines, using interpolation when data are not available. Euler poles (latitude, longitude, rotation angle) are calculated with 95 % confidence. Full squares represent average poles based on paleomagnetic poles within ± 5 Myrs of the age of interest. Dotted squares represent a best guess obtained using interpolation for time slices lacking data coverage. For comparison, the latitudinal position of Baltica in the reconstructions of ref.[6] is shown (red line). This alternative model is based on three input Euler poles (for 515, 443 and 425 Ma). The latitudinal evolution of Baltica is based on data from the 3 poles and a mix of paleobiogeography, paleoclimate and continental tectonics data as well as fundamental principles of plate tectonics (full framework described in ref.[7]). The degree of confidence in the latter reconstructions is evaluated in a semi-quantitative manner and approximates 53.5 % in the Ordovician[7]. Eq.: equator.

REFERENCES CITED

1. Song, H., Wignall, P. B., Song, H., Dai, X. & Chu, D. Seawater Temperature and Dissolved Oxygen over the Past 500 Million Years. *Journal of Earth Science* **30**, 236–243 (2019).

2. Goldberg, S. L., Present, T. M., Finnegan, S. & Bergmann, K. D. A high-resolution record of early Paleozoic climate. *Proceedings of the National Academy of Sciences* **118**, e2013083118 (2021).

3. Grossman, E. L. & Joachimski, M. M. Ocean temperatures through the Phanerozoic reassessed. *Scientific Reports* 1–14 (2022) doi:10.1038/s41598-022-11493-1.

4. Marcilly, C. M. *et al.* Understanding the early Paleozoic carbon cycle balance and climate change from modelling. *Earth and Planetary Science Letters* **594**, 117717 (2022).

5. Marcilly, C. M., Torsvik, T. H., Domeier, M. & Royer, D. L. New paleogeographic and degassing parameters for long-term carbon cycle models. *Gondwana Research* (2021) doi:10.1016/j.gr.2021.05.016.

6. Scotese, C. R. & Wright, N. PALEOMAP Paleodigital Elevation Models (PaleoDEMS) for the Phanerozoic (PALEOMAP Project, 2018). https://www.earthbyte.org/paleodem-resource- scotese-and-wright-2018/ (2018).

7. Scotese, C. R. *Plate Tectonics Flipbook, Van der Voo Retirement Symposium*. (2015).
