# Supplementary material for: Impact of global climate cooling on Ordovician marine biodiversity
Source: Nat Commun. 2023 Oct 10;14:6098. doi: 10.1038/s41467-023-41685-w (PMC10564867; doi:10.1038/s41467-023-41685-w)
Supplement: Supplementary file 3 — Reporting Summary [file 41467_2023_41685_MOESM3_ESM.pdf]

nature portfolio

Corresponding author(s): A. Pohl and D. Eliahou-Ontiveros

Last updated by author(s): Aug 16, 2023

Reporting Summary

Nature Portfolio wishes to improve the reproducibility of the work that we publish. This form provides structure for consistency and transparency in reporting. For further information on Nature Portfolio policies, see our Editorial Policies and the Editorial Policy Checklist.

Statistics

For all statistical analyses, confirm that the following items are present in the figure legend, table legend, main text, or Methods section.

n/a	Confirmed	
☒	☐	The exact sample size (n) for each experimental group/condition, given as a discrete number and unit of measurement
☒	☐	A statement on whether measurements were taken from distinct samples or whether the same sample was measured repeatedly
☒	☐	The statistical test(s) used AND whether they are one- or two-sided<br>*Only common tests should be described solely by name; describe more complex techniques in the Methods section.*
☒	☐	A description of all covariates tested
☒	☐	A description of any assumptions or corrections, such as tests of normality and adjustment for multiple comparisons
☐	☒	A full description of the statistical parameters including central tendency (e.g. means) or other basic estimates (e.g. regression coefficient) AND variation (e.g. standard deviation) or associated estimates of uncertainty (e.g. confidence intervals)
☒	☐	For null hypothesis testing, the test statistic (e.g. F, t, r) with confidence intervals, effect sizes, degrees of freedom and P value noted<br>*Give P values as exact values whenever suitable.*
☒	☐	For Bayesian analysis, information on the choice of priors and Markov chain Monte Carlo settings
☒	☐	For hierarchical and complex designs, identification of the appropriate level for tests and full reporting of outcomes
☒	☐	Estimates of effect sizes (e.g. Cohen's d, Pearson's r), indicating how they were calculated

Our web collection on statistics for biologists contains articles on many of the points above.

Software and code

Policy information about availability of computer code

Data collection	We used the software Matlab to conduct the macroecological model simulations. All material needed to repeat (and/or alter) the simulations is deposited on GitHub (see Data Availability statement): "All material required to generate the model results is available online and assigned a DOI: https://doi.org/10.5281/zenodo.8307366."
Data analysis	We used the software Matlab to conduct the analysis. We also used Yannick Copin's code for the Taylor Diagram of Fig. 3. Our code is deposited on GitHub and this information is provided in the manuscript (see Code Availability statement): "All material required to repeat the experiments, conduct the analysis of the results and generate the figures is available online and assigned a DOI: https://doi.org/10.5281/zenodo.8307366. The Matlab code71 is also available on GitHub (https://github.com/DanElie/Cooling-Oceans-Triggered-GOBE/tree/v1.2); this is the recommended mode of access as it will contain any updates and clarifications. Yannick Copin's code used for the Taylor Diagram in Fig. 3 is available on GitHub (https://gist.github.com/ycopin/3342888)."

For manuscripts utilizing custom algorithms or software that are central to the research but not yet described in published literature, software must be made available to editors and reviewers. We strongly encourage code deposition in a community repository (e.g. GitHub). See the Nature Portfolio guidelines for submitting code & software for further information.

Data

Policy information about availability of data

All manuscripts must include a data availability statement. This statement should provide the following information, where applicable:
- Accession codes, unique identifiers, or web links for publicly available datasets
- A description of any restrictions on data availability
- For clinical datasets or third party data, please ensure that the statement adheres to our policy

All material required to generate the model results is available online and assigned a DOI: https://doi.org/10.5281/zenodo.8307366. Biodiversity data used for model-data comparison were extracted from previously published work (see references where appropriate in the manuscript).

Research involving human participants, their data, or biological material

Policy information about studies with human participants or human data. See also policy information about sex, gender (identity/presentation), and sexual orientation and race, ethnicity and racism.

Reporting on sex and gender	Not applicable
Reporting on race, ethnicity, or other socially relevant groupings	Not applicable
Population characteristics	Not applicable
Recruitment	Not applicable
Ethics oversight	Not applicable

Note that full information on the approval of the study protocol must also be provided in the manuscript.

Field-specific reporting

Please select the one below that is the best fit for your research. If you are not sure, read the appropriate sections before making your selection.

☐ Life sciences ☐ Behavioural & social sciences ☒ Ecological, evolutionary & environmental sciences

For a reference copy of the document with all sections, see nature.com/documents/nr-reporting-summary-flat.pdf

Ecological, evolutionary & environmental sciences study design

All studies must disclose on these points even when the disclosure is negative.

Study description	Quantifying the impact of global climate cooling on marine biodiversity during the Ordovician. Conducting new (numerical) simulations, analyzing the results and comparing simulated trends with biodiversity curves taken from the literature.
Research sample	Biodiversity data used for model-data comparison were extracted from previously published work (see references where appropriate in the manuscript).
Sampling strategy	Extraction (digitalization) of biodiversity curves from the literature. No new sampling / data collection conducted here.
Data collection	Digitalization of biodiversity curves from published work by first author DEO (see references where appropriate in the manuscript).
Timing and spatial scale	Not applicable.
Data exclusions	No data collected / excluded.
Reproducibility	All files and code required to repeat the experiments are provided.
Randomization	No data collected.
Blinding	No data collected.

Did the study involve field work? ☐ Yes ☒ No

Reporting for specific materials, systems and methods

We require information from authors about some types of materials, experimental systems and methods used in many studies. Here, indicate whether each material, system or method listed is relevant to your study. If you are not sure if a list item applies to your research, read the appropriate section before selecting a response.

Materials & experimental systems

n/a	Involved in the study
☒	☐ Antibodies
☒	☐ Eukaryotic cell lines
☒	☐ Palaeontology and archaeology
☒	☐ Animals and other organisms
☒	☐ Clinical data
☒	☐ Dual use research of concern
☒	☐ Plants

Methods

n/a	Involved in the study
☒	☐ ChIP-seq
☒	☐ Flow cytometry
☒	☐ MRI-based neuroimaging
